# Gamma knife radiosurgery for the treatment of uveal melanoma and uveal metastases

**DOI:** 10.1186/s40942-017-0070-2

**Published:** 2017-05-29

**Authors:** Margaret M. Reynolds, Andrea L. Arnett, Ian F. Parney, Ravi Kumar, Nadia N. Laack, Patrick R. Maloney, Timothy F. Kozelsky, Yolanda I. Garces, Robert L. Foote, Jose S. Pulido

**Affiliations:** 1 Department of Ophthalmology, Mayo Clinic, 200 First Street, SW, Rochester, MN 55905 USA; 2 Department of Radiation Oncology, Mayo Clinic, Rochester, MN USA; 3 Department of Neurosurgery, Mayo Clinic, Rochester, MN USA; 4 Department of Molecular Medicine, Mayo Clinic, 200 First Street, SW, Rochester, MN 55905 USA

**Keywords:** Gamma knife, Intraocular metastasis, Stereotactic radiosurgery, Uveal melanoma, Uveal metastases

## Abstract

**Background:**

This study retrospectively analyzed outcomes for patients undergoing gamma knife radiosurgery (GKR) for uveal melanoma (UM) and intraocular metastases.

**Methods:**

Patients who underwent GKR for UM or intraocular metastases between 1/1/1990 and 6/1/2015 at Mayo Clinic, Rochester, MN, USA, were retrospectively analyzed.

**Results:**

Eleven patients (11 eyes) had UM while seven patients (7 eyes) had intraocular metastases. Patients with UM were followed for a median of 19.74 ± 10.4 months. Visual acuity (VA) logMAR 0.30 ± 0.53 (Snellen 20/40) versus 0.40 ± 0.97 (Snellen 20/50), tumor thickness (5.30 ± 2.17 vs. 3.60 ± 2.32 mm), were not significantly different between preoperative and postoperative measurements, respectively. Nine percent (1/11) patients required enucleation. Subsequently, no patients experienced metastases. Patients with intraocular metastases were followed for a median of 6.03 ± 6.32 months. They did not have significant changes in VA (logMAR 0.30 ± 0.59 vs. 0.30 ± 1.57; Snellen 20/40 vs. 20/40) or tumor thickness (3.50 ± 1.36 vs. 1.30 ± 0.76 mm) postoperatively. Fourteen percent (1/7 patients) required enucleation. Complications experienced by patients with UM include radiation retinopathy (2/11), papillopathy (1/11), cystoid macular edema (1/11), vitreomacular traction (1/11), exudative retinal detachment (1/11). Patients with metastases had treatment complicated by recurrence (2/7). Dose to the margin, maximum dose of radiation, and clinical target volume did not correlate with post-procedural VA, risk of enucleation, or death in patients with either UM or patients with intraocular metastases.

**Conclusions:**

Visual outcomes were satisfactory for patients undergoing GKR without significant morbidity and without significant risk of enucleation or metastases.

## Background

Ocular metastases are the most common intraocular malignancy, while uveal melanoma (UM) is the most common primary intraocular malignancy. Cancer treatments have evolved to prioritize the most effective minimally invasive treatments with the fewest side effects. For these reasons, proton beam therapy, plaque brachytherapy, and gamma knife radiosurgery (GKR) have become more common. Recent studies have demonstrated that GKR has a similar efficacy to proton beam therapy and plaque brachytherapy [1–19].

Patients with cancer are living longer, resulting in higher rates of ocular metastases [20]. Patients with known malignancy have an estimated incidence of ocular involvement at 4–12% in post-mortem studies [18, 21–23] and clinical apparent malignancies in 2.3–5% of patients [18, 21, 24]. The most prevalent metastases have been reported to be breast carcinoma and lung carcinoma, which make up 80% of cases [25, 26]. Patients with uveal metastases have a relatively short life expectancy, with a mean survival of 7 months [18]. Still, without treatment, the metastatic disease is typically progressive with a poor visual prognosis and high ocular morbidity [18]. The goal of treatment for patients with ocular metastases is to decrease the tumor burden and ocular morbidity. Therefore, ocular treatments with the most efficacy, shortest duration of treatment, and fewest side effects are prioritized. For these reasons, proton beam therapy, plaque brachytherapy, and GKR have replaced external beam radiation, which requires weeks of treatment with more side effects.

Uveal melanoma is the most common primary intraocular malignancy in adults with an incidence of 5.1 per million people [27]. Since the Collaborative Ocular Melanoma Treatment Study (COMS) revealed that conservative treatments, such as brachytherapy, had the same survival outcome as surgical treatment, i.e. enucleation, physicians have prioritized more conservative treatment with the goal of preserving vision and eyes in patients with UM [28]. Both GKR and proton beam therapy have similar outcomes as enucleation and are, therefore, both utilized for treatment of large UMs [28, 29]. The goal of radiotherapy is to conserve the eye, destroy the tumor, and prevent local recurrence.

With similar efficacy to proton beam therapy and plaque brachytherapy, GKR also has some advantages. Unlike plaque brachytherapy, which requires two procedures on separate dates—placing and removing a plaque, GKR is a same-day procedure. Proton beam therapy facilities are resource intensive and not universally available.

Few reports have been published describing the use of GKR in eyes with UM and uveal metastases. We wish to report our results of patients that underwent GKR for UM and uveal metastases.

## Methods

This study retrospectively analyzed patients with primary UM and uveal metastases who were treated with GKR at Mayo Clinic Rochester between 1/1/1990 and 6/1/2015. Approval was obtained from the Mayo Clinic Institutional Review Board.

Individuals considered for this study underwent GKR for choroidal metastases or primary UM. Patients were required to have at least one follow-up appointment. Data obtained from patient records included: date of birth, sex, oncologic diagnosis, preoperative and postoperative visual acuity (VA), tumor thickness, largest-base dimension (LBD), intraocular pressure (IOP), additional ophthalmic procedures such as enucleation, and post-procedural complications such as radiation retinopathy. Patients without follow-up and patients who underwent GKR for orbital rather than intraocular tumors were excluded. Patients were selected for GKR who were not candidates for plaque brachytherapy as they had melanomas, which were larger than the size of the largest plaque used for plaque brachytherapy (24 mm). GKR was chosen over enucleation after discussion with patients. During the period of this study, proton beam therapy was not yet available at Mayo Clinic, Rochester.

**Fig. 1:**
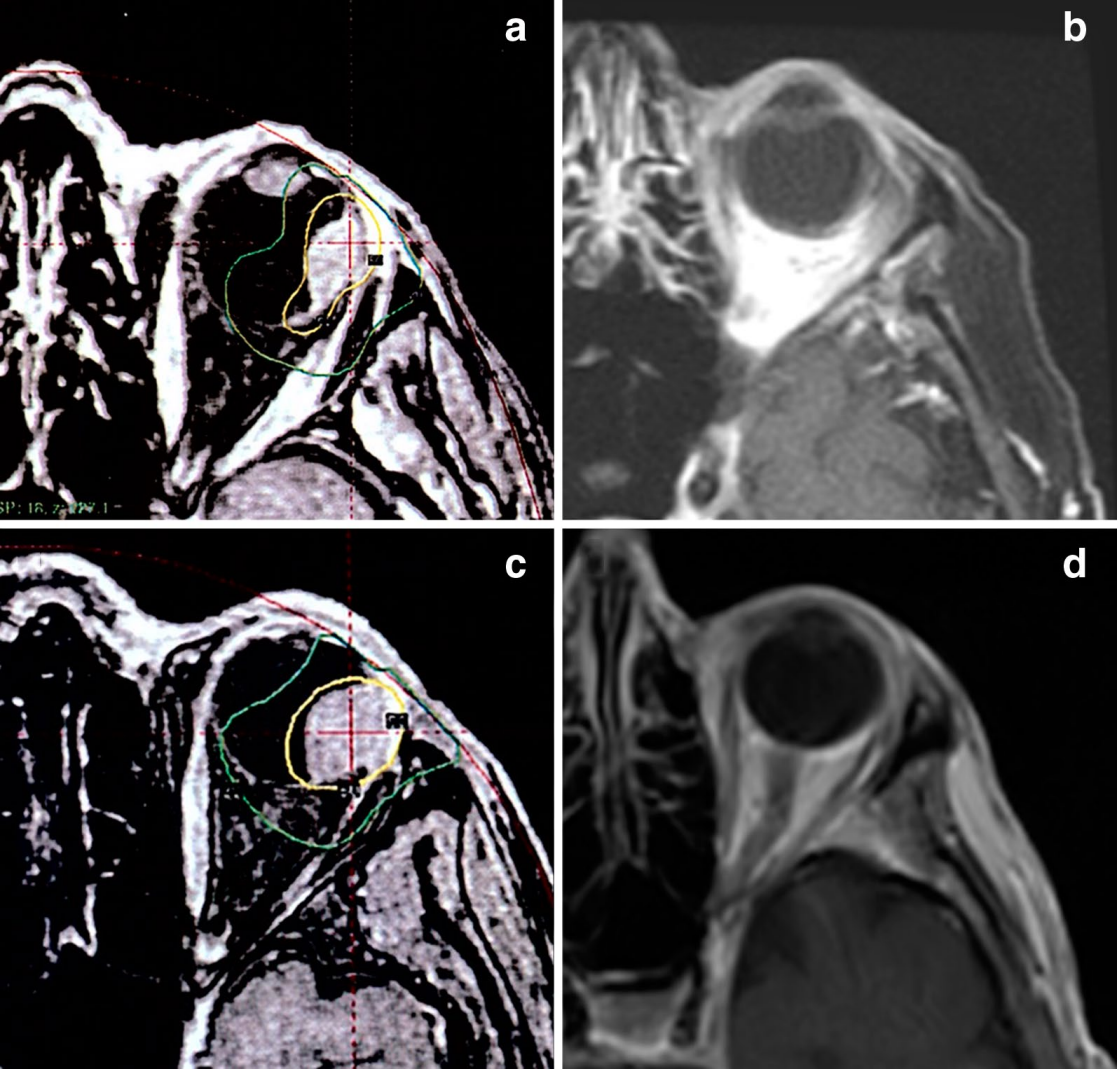
**a** Representative gamma knife planning MRI of a patient with choroidal melanoma treated with 18 Gy at the 50% isodose line. **b** gadolinium-enhanced T2 MRI of the orbit of the same patient depicted in tile **a**, after 7 months, shows interval decrease in size of choroidal mass. **c** Representative gamma knife planning MRI of a patient with choroidal melanoma treated with 27 Gy at the 50% isodose line. **d** Gadolinium-enhanced T2 fat saturation MRI of the orbit of the same patient depicted in tile **c**, after 48 months, shows interval decrease in size of choroidal mass

All included patients underwent GKR according to the following technique: retrobulbar block was performed. The Leksell stereotactic head frame was applied using local anesthetic and superficial fixation to the outer plate of the skull as described by Safaee et al. [30]. Patients underwent magnetic resonance imaging (MRI) with gadolinium contrast of the orbits and returned to the gamma knife center. Images were imported into the treatment planning system, Leksell GammaPlan, Elekta AB, Stockholm, Sweden. MRI and three-dimensional modeling was utilized to determine the tumor margins. A treatment plan was then developed in conjunction with a radiation oncologist, neurosurgeon, and ocular oncologist (Fig. 1). The clinical target volume (CTV) was determined to be the gross tumor volume (GTV) plus 2 mm on each side. The patient was transferred to the treatment unit, where the stereotactic GKR was performed. All patients were discharged the same day.

Categorical variables were compared between patients with uveal metastases and melanoma using the χ^2^ test, and two-sample t tests were used to analyze continuous patient characteristics. Correlation tests were used to compare radiation doses and visual outcomes. Simple logistic models were constructed to determine variables associated with increased odds of enucleation and death. Statistical analyses were conducted using commercial software JMP (SAS Institute, Cary, NC, USA). All statistical tests were two-sided with a 0.05 level of significance.

## Results

Eighteen patients met inclusion criteria; seven patients with uveal metastasis [lung adenocarcinoma (n = 2), breast adenocarcinoma (n = 1), renal cell carcinoma (n = 1), cystic carcinoma (n = 1), metastatic melanoma (n = 1), esophageal carcinoma (n = 1)] and 11 patients with primary UM.

Demographic characteristics are demonstrated in Table 1. Patients with UM were a median of 76.9 ± 10.0 years old. Patients had a median follow-up of 19.74 ± 10.4 (range 3.40–26.7) months. Of note, the three patients who succumbed to UM did so <6 months postoperatively. Patients who did not succumb to the illness were followed between 11.6 and 26.66 months (median: 22.1 ± 7.47 months). Patients with UM had tumors, which were a median of 5.30 ± 2.17 mm thick by ultrasound with a preoperative LBD of 19.9 ± 4.55 mm. Patients with UM were treated with a marginal dose of 25.0 ± 3.36 Gy at the 50% isodose line with a maximum median dose of 50.0 ± 8.61 Gy. The median CTV was 2250 ± 747 mm^3^ (Table 2). Eight of eleven patients (72.7%) were surviving at completion of the study. The final tumor thickness was 3.60 ± 2.32 mm, and postoperative LBD was 17.6 ± 1.90 mm (Table 1).

**Table 1:** Demographic characteristics of patients undergoing gamma knife radiosurgery

	Melanoma	Metastases
Age at gamma knife (years)	76.9 ± 10.0	59.0 ± 12.8
Female (%)	63.6	28.5
Length of follow-up (months)	19.74 ± 10.4	6.03 ± 6.32
Survival (%)	72.7	14.3
Tumor recurrence (% recurrence)	0	28.6
Pre-op tumor thickness by ultrasound (mm)	5.30 ± 2.17	3.50 ± 1.36
Post-op tumor thickness by ultrasound (mm)	3.60 ± 2.32	1.30 ± 0.76
Pre-op VA (logMAR/Snellen)	0.30 ± 0.53/20/40	0.30 ± 0.59/20/40
Pre-op IOP (mmHg)	14.0 ± 2.40	14.0 ± 7.31
VA at last follow-up (logMAR/Snellen)	0.40 ± 0.97/20/50	0.30 ± 1.57/20/40
IOP at last follow-up (mmHg)	15.0 ± 3.49	17.0 ± 3.39
Enucleation (%)	9.09	14.3
Largest base dimension pre-op (mm)	19.9 ± 4.55	14.0 ± 5.55
Largest base dimension post-op	17.6 ± 1.90	13.50 ± 9.62

**Table 2:** Gamma knife treatment parameters

	Melanoma	Metastases
Maximum dose of radiation (Gy)	50.0 ± 8.61	44.0 ± 5.52
50% isodose (Gy)	25.0 ± 3.36	20.0 ± 2.34
Clinical target volume (mm^3^)	2250 ± 747	1770 ± 2791

Of the 10 patients with UM who did not undergo enucleation, VA increased by two or more lines in 0% (0/10), was stabilized in 70% (7/10 eyes), and decreased in 30% (3/10) due cystoid macular edema, radiation retinopathy, and exudative retinal detachment. In patients with UMs, VA, IOP, LBD, and tumor thickness were not significantly different postoperatively (Table 3). Notably, the tumor thickness was less postoperatively 5.30 ± 2.17 versus 3.60 ± 2.32 mm, but this was not statistically significant (p = 0.07), perhaps due to small study size. Dose to the margin, maximum dose of radiation, and CTV did not correlate with post-procedural VA, IOP, risk of enucleation, or death in patients with UM.

**Table 3:** Preoperative versus postoperative demographic characteristics for patients with uveal melanoma and metastases

	Pre-op	Post-op	p value
Melanoma			
Visual acuity logMAR/Snellen	0.30 ± 0.53/20/40	0.40 ± 0.97/20/40	0.27
IOP	14.0 ± 2.40	15.0 ± 3.49	0.62
Tumor thickness (mm)	5.30 ± 2.17	3.60 ± 2.32	0.07
Largest base dimension (mm)	19.9 ± 4.55	17.6 ± 1.90	0.43
Uveal metastases			
Visual acuity logMAR/Snellen	0.30 ± 0.59/20/40	0.30 ± 1.57/20/40	0.76
IOP	14.0 ± 7.31	17.0 ± 3.39	0.92
Tumor thickness (mm)	3.50 ± 1.36	1.30 ± 0.76	0.01
Largest base dimension (mm)	14.0 ± 5.55	11.3 ± 9.62	0.58

Of the included patients with UM, 18% (2/11) experienced radiation retinopathy, 9% (1/11) underwent enucleation, 27% (3/11) had papillopathy, 9% (1/11) had cystoid macular edema, 9% (1/11) had vitreomacular traction, and 9% (1/11) had exudative retinal detachment (Table 4). Finally, one patient with UM underwent enucleation after the tumor did not respond and demonstrated growth post-procedurally.

**Table 4:** Complications experienced by patients undergoing gamma knife radiosurgery

	Melanoma	Metastases
Length of follow-up (months)	19.74 ± 10.4	6.03 ± 6.32
Radiation retinopathy	2/11	0/7
Vitreous hemorrhage	0/11	0/7
Neovascular glaucoma	0/11	0/7
Recurrence	0/11	2/7
Enucleation	1/11	1/7
Papillopathy	1/11	0/7
Cystoid macular edema	1/11	0/7
Vitreomacular traction	1/11	0/7
Exudative retinal detachment	1/11	0/7

Patients with metastases were a median of 59.0 ± 12.8 years. Patients had a median follow-up of 6.03 ± 6.32 (range 3.28–22.07) months. Tumor thickness was a median of 3.50 ± 1.36 mm with LBD of 14.0 ± 5.55 mm, preoperatively. Patients with metastasis were treated with a marginal dose of 20.0 ± 2.34 Gy at the 50% isodose line with a median maximum dose of 44.0 ± 5.52 Gy. The median CTV was 1770 ± 2791 mm^3^ (Table 2). Patients with uveal metastases had significantly decreased tumor thickness postoperatively (p = 0.01). They did not have significant changes in VA, IOP, or LBD postoperatively (Table 3).

One out of seven patients (14.3%) with uveal metastases was surviving at completion of the study. The final tumor thickness was 1.30 ± 0.76 mm with an LBD of 13.50 ± 9.62 mm (Table 1). One patient underwent enucleation for pain control. This patient presented with 10 out of 10 pain, presumed to be neuropathic in origin due to metastatic disease. The patient had macular degeneration in the eye not affected by metastasis with a VA of 20/50, so GKR was attempted to spare the eye. Due to persistence of pain, the patient elected for enucleation. Of the six patients with uveal metastases who did not undergo enucleation, visual acuity increased by two or more lines in 14.3% (1/7), was stabilized in 28.6% (2/7), and decreased in 57.1% (4/7). Two of seven patients with choroidal metastases had recurrence at the same location as the previously treated lesions in the eye, but no extraocular progression was attributable to the eye. Of the two patients with recurrence, one had adenoid cystic carcinoma. The other had esophageal adenocarcinoma. It is possible that these tumors were less responsive to radiotherapy, required a higher dose, or were more malignant.

Dose to the margin, maximum dose of radiation, and CTV did not correlate with post-procedural VA, IOP, risk of enucleation, or death in patients with uveal metastases.

Of the patients with metastatic disease, 28.6% (2/7) experienced local recurrence as defined by new choroidal lesions, and 14.3% (1/7) underwent enucleation for post-procedural neuropathic pain. Of note, this pain was present prior to GKR, but GKR was pursued instead of primary enucleation as the patient had macular degeneration and poor vision in the eye unaffected by the metastasis (Table 4).

## Discussion

This clinical investigation of GKR for patients with choroidal metastasis and UM found a positive correlation existed for patients with uveal metastases between marginal dose and post-procedural intraocular pressure. Marginal dose, maximal dose, and CTV did not correlate with post-procedural VA, risk of enucleation, or death in patients with either UM or patients with uveal metastases. Though the marginal dose and the 50% isodose were determined and recorded, we did not have the dose to the lens or retina on these cases. This study provides new insights into outcomes of patients with UM and uveal metastases treated with GKR.

For treatment of UM, both GKR and proton beam radiotherapy have been shown to have similar outcomes as enucleation [28, 29]. The COMS trial, which included more than 650 patients treated with plaque brachytherapy, found 88.7% of patients achieved local control with a recurrence rate of 10.3% and a survival rate of more than 80% at 5 years [31, 32].

Other studies on proton beam radiotherapy have demonstrated satisfactory outcomes. A prospective study by Gragoudas et al. reviewed 1922 consecutive patients treated with proton beam radiotherapy over 20+ years with an average follow-up for patients was 5.2 years. Ninety-seven percent of patients achieved local tumor control with a recurrence rate of 4.9% (45 patients). Seventeen patients required enucleation due to suspected tumor progression [33, 34].

A study by Modorati et al. [1] of 78 patients with tumor thickness, ranging from 3.1 to >10 mm over 12 years, treated with between 30 and 50 Gy (50% isodose) with GKR, found a survival rate of 88.8% at 3 years and 81.9% at 5 years, which was independent of dose. After treatment, 91% of patients had local tumor control with median tumor thickness reduced by 1.9 mm from a median baseline of 6.1 mm; 89.7% of patients avoided enucleation, although patients had significantly decreased vision after treatment (from 0.3 before treatment to an average VA of 0). Vision-compromising complications occurred such as exudative retinopathy (33.3%), neovascular glaucoma (18.7%), radiogenic retinopathy (13.5%), and vitreous hemorrhages (10.4%). Another study of single-fraction stereotactic radiosurgery of 23 patients applied 20–25 Gy (mean 21.7 Gy) and found 91% had local control. Three patients developed metastases in 121 months of follow-up, 61% of patients lost vision, 35% of patients maintained vision >20/200 [2]. The outcomes of our study are similar to these. Notably, our percent survival (63.6%) is greater likely attributable to the shorter length of follow-up. Table 5 contains a review of other published studies on GKR for UM.

**Table 5:** A review of previous studies using gamma knife radiosurgery for uveal melanoma

Study	Eyes	Radiation dose (Gy)	Follow-up (months)	Visual acuity		Complications							Survival (%)
				Before	After	Radiation retinopathy (%)	Radiation neuropathy (%)	Neovascular glaucoma (%)	Neovascularization	Radiation maculopathy (%)	Vitreous hemorhage	Enucleation (%)	
Modorati et al. [1] GKR	78	50 (n = 7) 40 (n = 21) 35 (n = 47) at 50% isodose	31.3 (median)	0.3 (0.05–0.8)	0 (0–0.05)	13.5	15.5	18.7					88.8% at 3 years and 81.9% at 5 years
Joye et al. [2] GKR	23	21.7 at 50% isodose	41.5 (median)	20/20-CF	20/20-NLP	8.7	13.0	17.4	8.7		8.7		91.3%
Marchini et al. [3] GKR	12	55 ± 10 Gy to 60–90% isodose	6 (median)	Not reported	Not reported	8.33	8.33	8.33		8.33			Not reported
Chan et al. [4] GKR	6	25 at 50% isodose	24 (median)	20/40–20/80	20/50-LP	16.7	16.7	16.7					100%
Dinca et al. [5] GKR	170	50–70 (n = 24) 45 (n = 71) 35 (n = 62) at 50% isodose	63.5 (median)	Patients with more than three lines decrease in VA at 5 years 45% (35 Gy), 89% (45 Gy), 93% (50–70 Gy)		25.81–41.67	12.68–20.83	8.06–20.82					5-year survival rates: 64% for 35 Gy, 62.71% for 45 Gy, 63.6% for 50–70 Gy
Sarici et al. [6] GKR	50	30 at 50% isodose	40 (median)	NR visual acuity decreased significantly after treatment (p < 0.0001)		24	14	14					87%
Sikuade et al. [7] GKR	85	35 at 50% isodose	39 (mean)	Not recorded	33% better than 6/60. 65% loss of ≥3 Snellen lines	NR							84%
Bellman et al. [8] GKR	5	50 maximum average dose	7.3 (median)	20/20–20/320	20/20-CF	NR							No deaths
Furdova et al. [9] GKR	96	49.0 maximum average dose	24 (mean)	16% ≥ 20/40, 58% < 20/40 ≥ 20/200, 26% < 20/200	11% ≥ 20/40, 47% < 20/40 ≥ 20/200, 42% < 20/200							11.5	Tumor local control was successful in 80% of patients in 5 years interval after stereotactic radiosurgery
Suesskind et al. [10] SDRT	60	25 at 50% isodose	33.7 (median)	20/20-LP	Median loss of −18 Snellen lines	42	24	15					83%
Kang et al. [11] GKR	22	45.6 at 50% isodose	67 (median)	LP to 1.2	NLP to 0.9	22.7		9.1					90.9%
Eibl-Lindner [12] frameless, single-session, image-guided robotic radiosurgery	217	20.3 at the 69% isodose	29.6 (mean)	A total of 104 patients presented with functional vision (defined as visual acuity ≥0.3) before treatment	Functional vision maintained in 30.9% of patients	13.4		15.2					Actuarial disease-specific survival was 84.8% (95% CI 77.0–90.1%) at 3 years and 78.4% (95% CI 67.1–86.2%) at 5 years
Haas et al. [13] GKR	32	50 at 50% isodose	38 (mean)	20% ≤ 20/400	78% ≤ 20/400	84		47					Metastasis free 94%, overall NR
Langmann et al. [14] GKR	60	50–70 isodose 50–80%	16–94 (range)	Not recorded	Not recorded		20	35					Metastasis free 85%, overall NR
Simonova et al. [15] GKR	81	31.4 minimum dose	32 (median)	Not recorded	Not recorded		12.3	25					70.4%
Fakiris et al. [16] GKR	19	40 isodose 50%	40 (median)	Not recorded	Not recorded		11	0					86% at 5 years
Zehetmayer et al. [17] GKR	62	45–70 isodose 50%	23.8 (median)	56.8% ≥ 20/200	21% ≥ 20/200	17.3	19.8	12.3					84% at 3 years
Current study GKR	11	25.0 ± 3.36 at 50% isodose	19.74 ± 10.4 (median)	20/30-CF	20/30-LP	18.2	9	0					72.7%

*NR* not recorded

Evaluating outcomes of different treatment methods for ocular malignancies is key in determining the most efficacious treatments with the least amount of morbidity. Table 6 lists a summary of previous studies. In a study which included 36 patients with uveal metastases who underwent plaque treatment, 27 (75%) received plaque brachytherapy as first-line treatment; 9 (25%) patients received plaque treatment as secondary therapy after the tumor failed to respond to external beam radiotherapy, chemotherapy, or hormone treatment [35]. Patients were treated for an average time of 86 h with a mean dose of 68.80 Gy to the apex and 235.64 Gy to the base. Over 11 months, 34 patients (94%) demonstrated regression. Five of six eyes receiving plaque brachytherapy as a second-line treatment were successfully salvaged. Three patients experienced radiation retinopathy, radiation papillopathy, or both (8%) at a mean of 8 months after treatment. Fifty percent of patients survived to completion of the study [35]. Other studies have demonstrated acceptable results [36]. While these results are satisfactory, plaque radiotherapy was not an option for the patients in this study, given the size of patients’ lesions. It is also true that GKR spared patients an additional procedure—plaque placement, required for plaque radiosurgery.

**Table 6:** A review of previous studies using radiotherapy (SRT, EBRT, GKR) for uveal metastases

Study	Eyes	Radiation dose (Gy)	Follow-up (months)	Visual acuity		Complications			Survival (%)
				Before	After	Radiation retinopathy (%)	Radiation neuropathy (%)	Neovascular glaucoma (%)	
Bellman et al. [8] Single-dose or fractionated SRT	10	12–20 Gy in a single dose or 30 Gy over 10 days to 50% isodose	6.5 (median)	20/26–20/320	20/26-LP	NR			NR
Bajcsay et al. [19] EBRT	24	46 Gy (maximum average dose)	24 (mean)	0.1–0.7	Improvement of two lines	3.5	0	0	4.2
Wiegel et al. [18] Radiotherapy	65	40 Gy in 20 fractions (50% isodose)	5.8 (median)	20/32	Visual acuity increased for two or more lines in 36%, stabilized in 50%, and decreased in 14%	1.5	1.5		18
Current study GKR	7	20.0 ± 2.34 (50% isodose)	6.03 ± 6.32 (median)	20/25–20/600	20/25-HM	0	0	14.3	14.3

Proton beam therapy has also been demonstrated to have satisfactory results [37, 38]. A retrospective study, which included 55 eyes of 49 patients who underwent two fractions of 14 cobalt gray equivalents, found that tumor regression occurred in 84% of patients and stability occurred in 14% of patients. Forty-seven percent of patients had vision that remained stable or improved. Post-proton therapy complications occurred in 29% of patients, including madarosis (28%), lid burns (17%), iris neovascularization and neovascular glaucoma (8%), cataract (11%), radiation maculopathy (19%), and radiation papillopathy (22%) [38]. During the dates of this study, proton therapy was not yet available at Mayo Clinic Rochester; therefore, patients ineligible for plaque therapy were treated with stereotactic radiosurgery.

A stereotactic radiosurgery study, which included ten patients with choroidal metastases, found that local tumor control was achieved in all eyes. Eight of ten patients had decreased tumor size. No significant side effects were noted in follow-up of 1–34 months [8]. While this study only included ten patients, it is notable that they did not experience significant side effects. A review of other studies which looked at radiation and uveal metastases is reviewed in Table 5.

Comparing the study published in this paper to those discussed, it had a higher rate of recurrence (28.6%). Notably, patients included in this study had more rare metastatic cancers (as opposed to breast or lung cancer) and a lower survival rate (28.6%). Weaknesses of this study included retrospective design, a small patient size, limited follow-up, and limited information regarding dose to the macula and optic nerve.

## Conclusions

In summary, GKR is a useful alternative to plaque brachytherapy and proton beam therapy. It is particularly useful for patients who cannot or prefer not to undergo the procedures required for plaque brachytherapy or for whose tumor sizes disqualify them. It is also useful for patients who do not have access to proton beam therapy, which is geographically limited.

## Abbreviations

COMS: Collaborative Ocular Melanoma Treatment Study
CTV: clinical target volume
GKR: gamma knife radiosurgery
GTV: gross tumor volume
IOP: intraocular pressure
LBD: largest-base dimension
MRI: magnetic resonance imaging
UM: uveal melanoma
VA: visual acuity
